# Visual Attention and Poor Sleep Quality

**DOI:** 10.3389/fnins.2022.850372

**Published:** 2022-06-02

**Authors:** Amirhussein Abdolalizadeh, Samaneh Nabavi

**Affiliations:** ^1^Interdisciplinary Neuroscience Research Program, Tehran University of Medical Sciences, Tehran, Iran; ^2^Students’ Scientific Research Center, Tehran University of Medical Sciences, Tehran, Iran; ^3^Department of Cognitive Neuroscience, Institute for Cognitive Science Studies (ICSS), Tehran, Iran

**Keywords:** sleep quality, sleep deprivation, attention network, visual search, MRI, superior longitudinal fasciculus, arcuate fasciculus, brain mapping

## Abstract

**Background:**

Sleep deprivation disrupts visual attention; however, the effects of chronic poor sleep quality on it are not understood. The dorsal attention network (DAN) and the ventral attention network (VAN) are involved in visual attention and search (VSA), with the DAN being important for the serial attention network and the VAN for parallel “pop-out” visual search.

**Objective:**

The aim of the study was to evaluate correlation of sleep quality with visual attention and search, functional, and tracts’ properties of the DAN and VAN.

**Materials and Methods:**

We recruited 79 young male subjects and assessed their sleep quality using the Pittsburgh Sleep Quality Index (PSQI), dividing subjects into poor sleepers (PSs) and good sleepers (GSs) based on a cutoff of 5. Daytime sleepiness, sleep hygiene, depression, and anxiety levels were also evaluated. We assessed VSA using a computerized match-to-sample (MTS) task. We extracted functional networks and tracts of the VAN and DAN and statistically assessed group differences in task performance and imaging covarying age, depression, and anxiety. An interaction model with MTS × group was also done on imaging.

**Results:**

In total, 43.67% of subjects were PSs. Sleep quality significantly correlated with daytime sleepiness, sleep hygiene, depression, and anxiety (all *p* < 0.001). No between-group differences were seen in task performance and functional or tract properties of the attention networks. Interaction analysis showed that the task performance was highly reliant on the DAN in PSs and on the VAN in GSs.

**Conclusion:**

Our findings show no association between sleep quality and VSA in task performance and imaging correlates of the attention network. However, unlike the GS group, poor sleep quality is associated with VSA being more reliant on the DAN than on the VAN.

## Introduction

Proper sleep is required to maintain optimal cognitive function. Lack of nighttime sleep, even for one night, has detrimental effects on several cognitive functions, especially sustained attention and vigilance, but other cognitive aspects are also involved, including executive function and sensory perception (e.g., visuospatial perception) (Killgore, 2010). Evaluating different aspects of sleep (subjective quality, duration, daytime sleepiness, etc.) over a period of time, rather than simply assessing the duration of one-night sleep, can provide a more robust and general assessment of sleep function and quality. The Pittsburgh Sleep Quality Index (PSQI) (Buysse et al., 1989) is based on the aforementioned factors to assess sleep over a period of 1 month to better determine the presence of any chronic poor sleep quality. Using the PSQI and depending on the community studied, chronic poor sleep has been found in around one-third to half of the population (Buysse et al., 2008; Del Brutto et al., 2016; Hinz et al., 2017). Of great importance, poor sleep quality is usually associated with concurrent medical or psychiatric conditions (e.g., depression and anxiety) (Ramsawh et al., 2009; Huang and Zhu, 2020). Despite the high prevalence of chronic poor sleep quality and its associations with psychiatric comorbidities, its effects on cognition have not been extensively studied. Previous studies have shown that poor sleep quality based on the PSQI is associated with problems in sustained attention (Gobin et al., 2015; Siddarth et al., 2021), executive function (Rana et al., 2018), and working memory (Xie et al., 2019) in healthy community samples. However, complex cognitive functions, such as visuospatial attention and search, which require the proper function of multiple cognitive aspects (including but not limited to attention and visual pattern recognition), have not been studied.

Corbetta and Shulman (2002) proposed a dual network model for attention: the dorsal attention network (DAN) connecting visual and parietal areas (especially intraparietal sulcus [IPS]) to frontal areas (especially frontal eye fields [FEFs]), and the right-side dominant ventral attention network (VAN) connecting middle and superior temporal gyri and temporoparietal junction (TPJ) to the middle and inferior frontal areas. According to their model, the DAN is involved in orienting attention overtly or covertly in space. The VAN seems to work as a “circuit breaker” for the DAN, in which it directs the DAN to unattended task-related or unrelated salient stimuli (Corbetta and Shulman, 2002; de Schotten et al., 2011; Geng and Mangun, 2011; Geng and Vossel, 2013; Vossel et al., 2013). Of course, these two networks are highly connected having reciprocal connections, and the DAN also updates the VAN regarding the expected stimuli (Vossel et al., 2013). Importantly, the VAN is different from the salience network, in which the latter involves the cingulum, the pre-motor supplementary, and the insula–frontal operculum (Uddin, 2015). Tractography studies using diffusion-weighted imaging (DWI) have identified major tracts associated with each network. The superior longitudinal fasciculus (SLF) has three branches: SLF-1 located dorsally connecting the IPS to superior frontal areas and FEFs (i.e., nodes of the DAN), SLF-3 located ventrally connecting nodes of the VAN, and SLF-2 seems to connect lower parietal areas and the TPJ to superior frontal areas, probably being the pathway between the DAN and VAN (Hattori et al., 2018; Allan et al., 2019, 2020). Also, the arcuate fasciculus (AF) on the right side is involved in connecting the VAN nodes to each other (Bernard et al., 2020). However, how do these networks relate to visual search?

Visual search is a complex behavior requiring working memory, scenery-related information processing (e.g., pattern recognition, color, and shape), and attention. Two models are proposed for visual search. In the “serial” search (inefficient), the subject screens each object independently, matching it with the sample presented or asked. However, it seems that, in real-life situations, we usually implement the “parallel” strategy (efficient, “pop-out”); despite being overtly attended to one location in space, the attention is covertly scanning the periphery and nearby, searching for the target stimuli, and changing the overt attention toward the target upon finding it (Wolfe, 2020). In a previous study by Leonards et al., fMRI activation maps of both parallel and serial search strategies overlapped significantly on the IPS, FEFs, and occipital areas (e.g., lateral occipital cortex). Contrasting search strategies against each other revealed more activation in superior frontal areas in serial search than in parallel search (Leonards et al., 2000). In another study, the same patterns of serial vs. parallel activation differences were seen in frontal, superior parietal, and superior occipital regions (Kim et al., 2012). Although these two studies have not mapped the found areas on the networks, it seems the serial vs. parallel contrast mostly overlaps over the DAN. In a recent study by Ischebeck et al. (2021) using a designed task to force the subjects to use the overt serial visual search task resulted in higher activation of the DAN. In another study using an exploratory visual search task similar to real-life situations, DAN activation was associated with orienting toward the salient stimuli. By contrast, the right supramarginal gyrus, a major node in the VAN, was activated by processing the targets, probably indicating the template-matching function in this region (Macaluso and Ogawa, 2018). Direct intracranial recordings also support the notion that parallel search strategies are reliant on the VAN (Slama et al., 2021). Moreover, inactivating DAN nodes (FEFs and IPS) using repetitive transcranial magnetic stimulation disrupted the non-pop-out search strategy, while it had no effect on the pop-out visual search, proving a causal role of the DAN in the serial visual search (Wang et al., 2020).

In this study, we first aimed to examine the effects of chronic poor sleep quality on a complex attention task, visual search and attention, controlling for major confounds such as depression and anxiety. Next, we examined the effects of chronic poor sleep quality on neuroimaging correlates of visual attention using fMRI to identify the DAN and the VAN and using DWI to delineate fibers connecting these two networks (i.e., SLF 1-2-3 and right AF). Finally, using an interaction model, we attempted to identify the association between sleep quality × brain findings and task outcomes.

## Materials and Methods

### Subjects

In total, 79 subjects were recruited via local flyers and online advertisements using Twitter and Telegram channels in the city of Tehran, Iran [mean age (SD) = 24.10 (4.16)]. The inclusion criteria for this study were being men, being 20–40 years of age, having no current or previously untreated psychiatric or neurologic disorders, with no prior history of head trauma leading to loss of consciousness, with no history of claustrophobia, and with no current signs or symptoms of or admission history due to COVID-19. This study was approved by the Ethics Review Board of Iran University of Medical Sciences, Tehran, Iran, with the ID IR.IUMS.REC.1400.026.

### Sleep Questionnaires

Subject demographics including age, handedness, education, marital status, smoking behavior, and any drugs taken or discontinued were acquired. To assess sleep, we used the Persian-translated and validated versions of the Pittsburgh Sleep Quality Index (PSQI) (Buysse et al., 1989; Farrahi Moghaddam et al., 2012), Epworth Sleepiness Scale (ESS) (Johns, 1991; Sadeghniiat Haghighi et al., 2013), and Sleep Hygiene Index (SHI) (Mastin et al., 2006; Chehri et al., 2016). The PSQI includes 10 questions, some with several parts, and assesses sleep quality using seven components: subjective sleep quality, sleep latency, sleep duration, habitual sleep efficiency, sleep disturbances, use of sleep medications, and daytime dysfunction. The total PSQI score is the sum of the seven components, and values > 5 are considered to indicate poor overall sleep quality during the last month (Buysse et al., 1989, 2008). The subjects were categorized based on their PSQI total score: poor sleeper (PS; PSQI total > 5) vs. good sleeper (GS; PSQI total ≤ 5). The ESS evaluates daytime sleepiness by the probability of falling asleep during daytime. A higher ESS total score shows higher sleepiness. The SHI is a 13-item questionnaire targeted at activities reducing the quality or probability of initiating night-time sleep (e.g., use of caffeinated beverages before sleep and sleeping in an uncomfortable bedroom). A higher SHI score indicates poor sleep hygiene.

### Psychiatric Questionnaires

Depression and anxiety are major contributors and are also affected by sleep quality. We used Beck Depression Inventory II (BDI-II) (Beck et al., 1988, 1996; Ghassemzadeh et al., 2005) to evaluate depression; a higher sum of all question scores in this 21-item self-report questionnaire indicates higher depressive scores. We also used the State and Trait Anxiety Inventory (STAI) (Spielberger, 2010) to assess current (state; STAI-S) and trait (STAI-T) anxiety of the subjects. Higher scores indicate higher anxiety. A meta-analysis also supported that the STAI is the best anxiety questionnaire to examine anxiety in sleep problems (Pires et al., 2016).

### Visual Search and Attention

We used the match-to-sample (MTS) task of the Cambridge Neuropsychological Test Automated Battery (CANTAB) for visual search and attention (Robbins et al., 1994). In this computerized task, subjects were presented with a complex figure in the middle of the screen. Then, a few patterns were shown in the periphery, from which one was matched with the presented pattern. In the first trials, two patterns were presented in the periphery, and it was increased to eight patterns in the final trials. A total of 48 trials were conducted for each subject. Total correct, mean reaction time (RT), and mean RT change from 2 to 8 pattern trials were calculated for each participant. Prior to the task, the subjects were instructed by an expert cognitive scientist in CANTAB and were allowed to take mock trials to ensure they have completely learned the task.

We chose this task for several reasons: First, it can be easily performed irrespective of subjects’ education or prior experience. Second, it can assess two complex functions, namely, visual search and visual attention, at the same time. Third, in many tasks, the subjects’ hand movement time cannot be separated from the reaction time; however, in the MTS of CANTAB, the subjects had to hold the mouse button until they have found a match, release it, and touch the target on a touch screen. Thus, the interval between stimulus presentation and mouse click release is considered the reaction time. Of note, it is different from the delayed match-to-sample task, in which working memory load becomes increasingly important. Since previous studies have shown the association between working memory dysfunction and poor sleep quality (Xie et al., 2019), we chose the current task version to minimize the effects of working memory on task performance. Finally, the samples and stimuli were matched or non-matched based on their complex figures and different colors, resulting in a higher load on visual processing.

### Imaging Acquisition

All imaging acquisitions were carried out in a single session using a 3T Siemens Prisma MRI scanner located at the National Brain Mapping Laboratory (NBML), Tehran University, Tehran, Iran. The imaging protocols were as follows: T1 MPRAGE (TR/TE/TI: 2000/3.62/845 ms, FoV: 256 mm, voxel size: 0.8 mm^3^ × 0.8 mm^3^ × 0.8 mm^3^), T2 SPACE (TR/TE: 3200/409 ms, FoV: 256 mm, voxel size: 0.8 mm^3^ × 0.8 mm^3^ × 0.8 mm^3^), resting-state fMRI (TR/TE: 2000/30 ms, FoV: 240 mm, 180 measurements, voxel size: 3 mm^3^ × 3 mm^3^ × 3 mm^3^), diffusion MRI (TR/TE: 8000/92 ms, FoV: 220 mm, voxel size: 2 mm^3^ × 2 mm^3^ × 2 mm^3^, b-value: 1,000 s/mm^2^ in 64 directions, 3 b0s, phase-encoding direction: AP), and 3 b0s with opposite phase-encoding direction from the main DWI sequence (phase-encoding direction: PA). To minimize the possibility of subjects falling asleep under the scanner, which can affect our functional data, we acquired the resting-state fMRI sequence with subjects with open eyes and the third protocol (after localizer and T1 MPRAGE) with a duration of 6:08 mins.

### Diffusion-Weighted Imaging Analysis and Tractography

We used MRtrix for DWI preprocessing (Tournier et al., 2019). First, the DWI scan was denoised (Veraart et al., 2016), and Gibbs ringing artifact was removed (Kellner et al., 2016) and then preprocessed with *dwifslpreproc* command using reverse-phase encoding b0 scans to perform inhomogeneity distortion correction (Andersson et al., 2003; Smith et al., 2004; Andersson and Sotiropoulos, 2016). Then, B1 field inhomogeneity correction was performed using the ANTs algorithm (Tustison et al., 2010). Finally, the resulting preprocessed DWI scans were fed to the TRActs Constrained by Underlying Anatomy (TRACULA) tractography pipeline by FreeSurfer v 7.2.0 (Yendiki et al., 2011; Fischl, 2012; Maffei et al., 2021).

**FIGURE 1:**
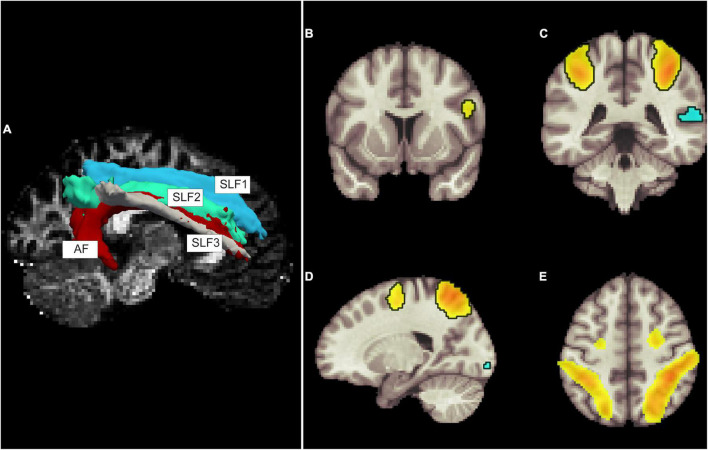
Tractography **(A)** and resting fMRI components of dorsal and ventral attention systems (DAN and VAN, respectively; **B–E**). **(A)** Tractography showing four tracts of interest on the right side including the three branches of superior longitudinal fasciculus (SLF) and arcuate fasciculus (AF). Figures b to e show the spatial components of the independent component analysis result identified as DAN and VAN. Coronal views showing **(B)** a region in the inferior frontal gyrus, **(C)** the bilateral intraparietal sulci and the supramarginal gyrus, **(D)** the lateral view showing spatial components in the superior frontal and intraparietal sulcus, as well as a small component in the occipital region, and **(E)** a superior view highlighting bilateral intraparietal sulci and superior frontal gyri. Superior frontal and intraparietal sulci are DAN nodes, and right-side dominant VAN can be visualized as spatial components in the right inferior frontal gyrus and the superior marginal gyrus.

Prior to tractography, the high-resolution structural scans (T1 and T2) were fed into the *recon-all* pipeline to extract white matter and pial surfaces. Cortical parcelation and subcortical volumetry were required for TRACULA. We also performed thalamic nuclei segmentation, which was suggested by Yendiki et al. (Maffei et al., 2021), to improve tractography for fibers passing the nearby thalamus (Iglesias et al., 2018). The default configuration settings text file of TRACULA was used with changing all preprocessing steps to zero (not perform). TRACULA transforms each subject’s DWI to an anatomical scan (affine transformation), extracts the fractional anisotropy (FA) map, non-linearly transforms it to a high-resolution FA map (named MGH35_HCP_FA_template.nii.gz), fits the probabilistic diffusion model using BedpostX (Behrens et al., 2003, 2007; Jbabdi et al., 2012), and then performs tractography. We selected the fibers connecting the DAN and VAN, both intra- and inter-network fibers, including bilateral SLFs (I-II-III) and right AF (Figure 1). The power of TRACULA lies in achieving connectome scanner-level accuracy and tractography quality with DWI data acquired with lower b-values and the number of diffusion directions (Maffei et al., 2021). We extracted the FA map and mean, axial, and radial diffusivity (MD, AD, and RD, respectively) for each tract afterward.

### fMRI Analysis

We used the default fMRI pipeline analysis of CONN toolbox v20.b^1^ (RRID:SCR_009550) (Whitfield-Gabrieli and Nieto-Castanon, 2012) using resting-state fMRI analysis. Functional data were first resampled to 2 mm^3^ × 2 mm^3^ × 2 mm^3^ voxel size, motion-corrected, centered, and slice timing-corrected. Outlier detection using ART was used with a global signal Z-value threshold of 5, and the subject displacement threshold was set to 0.9 mm. Then, the functional and structural data were segmented and normalized to the MNI space. We spatially smoothed the fMRI data using a 6 mm isotropic full-width at half maximum (FWHM) Gaussian kernel. The results of preprocessing were inspected individually to have structural and functional data aligned and normalized. We applied fMRI denoising using the CompCor method (Behzadi et al., 2007) and regressed out physiological noise sources with white matter, cerebrospinal fluid, and scrubbing and motion parameters. Linear detrending and a band-pass filter of 0.008–0.09 Hz were also applied.

To identify the DAN and VAN, we applied the group independent component analysis (ICA) tool in CONN. We estimated the median number of components of all subjects’ rest fMRI data to be 16 using GIFT toolbox v 3.0c^2^ (Calhoun et al., 2001). Group-level dimensionality reduction was set to 64, the number of factors set to 16, and G1 fast ICA using the group ICA 3 (GICA3) back-propagation method was implemented. Then, we computed spatial match to the template with CONN’s summary tools to identify networks. Using the spatial overlap of suprathreshold areas (Dice coefficient) resulted in a match with the DAN in one of the independent components, with *r* = 0.56 and bilateral IPS and FEFs involved. The VAN is not defined in the template of CONN; however, the same component identified as the DAN also included regions of the right inferior frontal and superior temporal/lower parietal areas (i.e., TPJ), especially on the right side. So, we considered the spatial regions of this component to include both attention networks, DAN and VAN (i.e., IPS, FEFs, right inferior frontal, supramarginal and superior temporal regions, and lateral occipital cortices) (Figure 1).

To evaluate between-group differences, we used a general linear model (GLM) with age, depression, and anxiety scores (both state and trait) as covariates in the identified spatial component of the VAN and DAN. Moreover, group × MTS score interaction analysis was also performed with the same covariates in the same networks to find group-related differences in behavior and baseline function correlates. Results with a voxel *p*-value threshold < 0.001 and a cluster-wise FDR-corrected *p* < 0.05 were considered significant.

### Statistical Analysis

We used R version 4.0.4 embedded within RStudio (R Core Team, 2021). Between-group differences (PS vs. GS) in age, BDI, and STAI scores were statistically assessed using the t-test or Mann–Whitney *U* test based on the distribution of data. We assessed the correlation between the PSQI total score and components with ESS, SHI, BDI, STAI-T, and STAI-S using Pearson’s or Spearman’s formula based on data distribution. We also used the GLM to assess any association between PSQI components and MTS scores, irrespective of the group, and adjusting for age and scores of depression and anxiety.

Differences in fiber properties of SLFs and AF between PSs and GSs were evaluated using the same GLM, with age, BDI, and STAI scores as covariates. We also applied an interaction model with MTS measures as the outcome and group × fiber properties as the predicting variable covarying age, BDI, and STAI scores.

## Results

### Subject Characteristics

**FIGURE 2:**
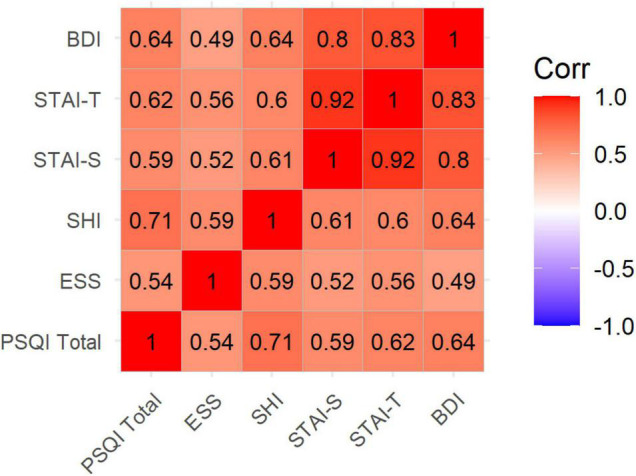
Correlation plot showing bivariate correlations between sleep assessments, depression, and anxiety scores. Correlation coefficients are written inside the cells. All *p*-values are <0.001. PSQI, Pittsburgh Sleep Quality Index; ESS, Epworth Sleepiness Scale; SHI, Sleep Hygiene Index; STAI, State and Trait Anxiety Inventory (STAI-T: Trait, STAI-S: State); BDI, Beck Depression Inventory.

The mean (SD) of the education years of the subjects was 12.20 (0.34). Of the 79 subjects, total PSQI scores could be calculated for 71 subjects; 31 subjects had a PSQI total score > 5, indicating a poor sleep quality prevalence of 43.67% in our sample. Although there is no consensus regarding the BDI-II cutoff for depression, using the suggested cutoff of 13 (BDI total ≥ 14) (Beck et al., 1996) indicates a prevalence of probable depression in our subjects of 34.67% (26 of 75 completed BDI scores). PS vs. GS differences showed significantly higher ESS, SHI, BDI, and anxiety scores (STAI-T and STAI-S) in PSs than in GSs (Table 1). Correlation analysis also showed that higher PSQI scores are significantly correlated with higher ESS, SHI, BDI, and anxiety scores (Figure 2).

**TABLE 1:** Mean (SD) of age, Epworth Sleepiness Scale, Sleep Hygiene Index, Beck Depression Inventory, and State and Trait Anxiety Inventory for each sleep quality group based on a Pittsburgh Sleep Quality Index (PSQI) total score of 5.

	Good sleep *n* = 40	Poor sleep *n* = 31	*p*-Value
Age^†^	24.86(5.25)	22.97(2.49)	0.32
Education^†^	12.25(0.63)	12.13(0.50)	0.30
Epworth Sleepiness Scale^†^	5.00(3.62)	8.23(4.33)	<0.001^***^
Sleep Hygiene Index	10.68(6.74)	20.72(7.43)	<0.001^***^
Beck Depression Inventory^†^	7.78(7.73)	19.11(13.72)	<0.001^***^
**State and Trait Anxiety Inventory**			
State^†^	36.10(9.91)	47.74(11.09)	<0.001^***^
Trait^†^	34.15(8.69)	45.77(10.37)	<0.001^***^

*† Distribution in either of the groups did not follow the normality (Shapiro–Wilk p < 0.05), so a non-parametric statistical test was used.*

^***^p-value < 0.001.

Of the 79 subjects, 20 right-handed PS and 19 right-handed GS (*n* = 39) were randomly matched by MatchIt (Ho et al., 2011) based on age for imaging and MTS task performance at the National Brain Mapping Laboratory, Tehran University, Tehran, Iran. Since there was a 3-month delay between questionnaire filling and MRI/MTS task sessions due to the COVID-19 Alpha/Delta variant surge in Iran, PSQI, BDI, and anxiety questionnaires were filled out again by the participants on the day of MRI acquisition. The new PSQI scores resulted in one of the GS subjects being transferred into our PS group (21 PSs and 18 GSs).

### Task Performance

Overall, 34 subjects successfully completed the MTS task (16 PSs and 18 GSs). While using a GLM with age, BDI, and STAI parameters as covariates, there were no significant associations between MTS task parameters (total correct, mean RT, mean RT change) and PSQI scores (total and each component) (Supplementary Table 1). Group differences are shown in Table 2.

**TABLE 2:** Mean (SD) of match-to-sample (MTS) visual search and attention task scores of CANTAB, and demographics for the selected subjects for imaging and cognitive evaluation session.

	Good sleep *n* = 18	Poor sleep *n* = 16	*p*-Value
**Demographics**			
Age	23.44(2.81)	22.75(2.24)	0.435
Education	12.15(0.38)	12.10(0.45)	0.722
BDI total	9.06(6.94)	20.06(10.48)	0.001
STAI-S	37.28(10.96)	46.44(9.03)	0.013
STAI-T	36.39(10.04)	44.50(7.83)	0.014
**MTS**			
Total correct	46.50(1.89)	46.25(1.73)	0.484
Percent correct	96.88(3.93)	96.35(3.61)	0.484
Mean correct MT (ms)	1,180.53(498.44)	989.39(418.92)	0.179
Mean correct RT (ms)	1,613.36(645.98)	1,562.58(713.89)	0.959
Mean error MT (ms)	1,696.68(940.08)	1,532.27(1018.00)	0.090
Mean error RT (ms)	2,002.61(1134.71)	1,952.81(1161.04)	0.895
Mean RT change [2–8 (ms)]	1,758.52(1181.04)	1,535.34(945.90)	0.742
Mean MT change [2–8(ms)]	970.52(1059.52)	661.71(707.67)	0.213

*p-values are calculated in a general linear model with age, depression, and anxiety scores as covariates.*

*BDI, Beck Depression Inventory; STAI-S/T, State and Trait Anxiety Index–state/trait; RT, reaction time; MT, movement time.*

### Between-Group Differences in Dorsal Attention Networks and Ventral Attention Networks Imaging

Without any prior history of claustrophobia or any fears of the dark or closed spaces, 4 subjects, all in the PS group, developed claustrophobia either at the beginning or even prior to the start of the T1 acquisition (during the localizer sequence). Overall, 35 subjects completed the MRI session.

After adjusting for age, depression, and anxiety scores, no differences were found between PS and GS in fiber properties of bilateral SLFs and right AF (Table 3). Moreover, there were no differences in the VAN and DAN between our groups (T-map available at https://identifiers.org/neurovault.collection:11254).

**TABLE 3:** Mean (SD) for fiber properties of superior longitudinal fasciculus (SLF) branches and right arcuate fasciculus (AF).

Fiber parameter	Good sleep *n* = 18	Poor sleep *n* = 17	*p*-Value
**Right SLF1**			
FA	0.523(0.027)	0.533(0.018)	0.604
AD	0.00119(4.118*e*−05)	0.00118(3.385*e*−05)	0.125
MD	0.00073(2.697*e*−05)	0.00072(1.784*e*−05)	0.058
RD	0.00050(3.062*e*−05)	0.00048(2.013*e*−05)	0.151
**Right SLF2**			
FA	0.416(0.025)	0.423(0.020)	0.088
AD	0.00108(2.271*e*−05)	0.00107(2.411*e*−05)	0.936
MD	0.00073(2.380*e*−05)	0.00072(1.548*e*−05)	0.187
RD	0.00056(2.928*e*−05)	0.00054(1.943*e*−05)	0.091
**Right SLF3**			
FA	0.444(0.025)	0.443(0.015)	0.759
AD	0.00108(2.122*e*−05)	0.00107(2.736*e*−05)	0.128
MD	0.00071(1.931*e*−05)	0.00071(1.470*e*−05)	0.213
RD	0.00053(2.584*e*−05)	0.00053(1.503*e*−05)	0.574
**Left SLF1**			
FA	0.531(0.022)	0.539(0.022)	0.943
AD	0.00118(4.346*e*−05)	0.00118(4.553*e*−05)	0.286
MD	0.00072(2.898*e*−05)	0.00071(1.943*e*−05)	0.169
RD	0.00049(2.914*e*−05)	0.00048(2.086*e*−05)	0.214
**Left SLF2**			
FA	0.412(0.025)	0.412(0.017)	0.976
AD	0.00108(2.907*e*−05)	0.00106(2.316*e*−05)	0.233
MD	0.00073(2.715*e*−05)	0.00072(1.462*e*−05)	0.336
RD	0.00056(3.204*e*−05)	0.00055(1.771*e*−05)	0.550
**Left SLF3**			
FA	0.460(0.024)	0.461(0.020)	0.982
AD	0.00110(2.936*e*−05)	0.00108(3.290*e*−05)	0.137
MD	0.00072(2.630*e*−05)	0.00070(1.916*e*−05)	0.167
RD	0.00053(3.003*e*−05)	0.00051(1.936*e*−05)	0.353
**Right AF**			
FA	0.435(0.020)	0.440(0.018)	0.464
AD	0.00111(2.385*e*−05)	0.00110(2.590*e*−05)	0.448
MD	0.00073(2.233*e*−05)	0.00072(1.448*e*−05)	0.255
RD	0.00054(2.553*e*−05)	0.00054(1.726*e*−05)	0.291

*FA, fractional anisotropy; MD, mean diffusivity; AD, axial diffusivity; RD, radial diffusivity.*

### 3.4 Interaction Analysis

**FIGURE 3:**
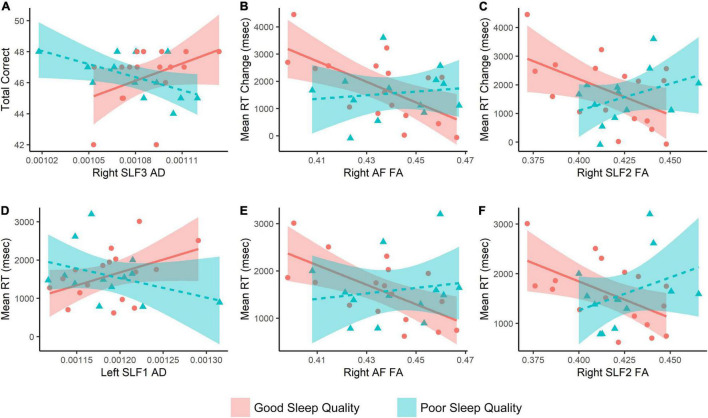
Interaction analysis results with match-to-sample scores as the outcome, and fiber properties × Group as the predictor variable. Age, depression, and anxiety scores are covaried. RT, reaction time; SLF, superior longitudinal fasciculus; AF, arcuate fasciculus; FA, fractional anisotropy; AD, axial diffusivity.

**FIGURE 4:**
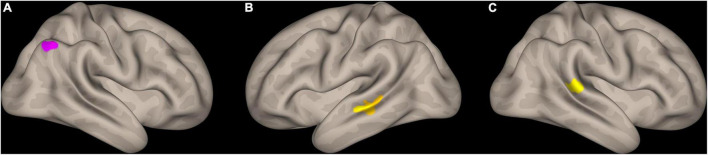
Interaction analysis results with match-to-sample scores as the outcome, and group × rest fMRI signal of the spatial components of dorsal and ventral attention systems identified through independent component analysis. **(A)** Cluster identified for mean correct RT × group (PS > GS) in the right lateral occipital cortex, in proximity to intraparietal sulcus, **(B)** cluster identified for total correct × group (GS > PS) in the left middle temporal gyrus (posterior division) and the superior temporal sulcus, **(C)** cluster identified for total correct × group (GS > PS) in the right superior marginal gyrus (posterior division). RT, reaction time; GS: good sleep; PS, poor sleep.

Overall, 30 subjects (13 PSs and 17 GSs) could successfully complete both MRI and MTS tasks. We found significant group × MTS measure interactions for MTS total correct and right SLF3 AD, MTS mean correct RT with left SLF1 AD, right SLF2 FA, right AF FA, MTS mean RT change with right AF FA, and right SLF2 FA (Supplementary Table 2 and Figure 3). Moreover, three significant clusters were also found for the interaction model (Table 4 and Figure 4). The results of the fMRI interaction analysis are uploaded on NeuroVault (T-maps on https://identifiers.org/neurovault.collection:11254).

**TABLE 4:** Results of fMRI group–ICA spatial component interaction models corrected at voxel level with uncorrected *p*-value < 0.001 and cluster size *p*-FDR < 0.05.

Interaction model	Coordinates (x, y, z; MNI)			Cluster size	Location (include most voxels)
Total correct × Group (Good > Poor)	–48	–20	–8	108	Left middle temporal gyrus (posterior division)
	+64	−36	+10	69	Right supramarginal gyrus (posterior division)
Mean correct reaction Time × Group (Poor > Good)	+48	–62	+52	86	Right lateral occipital cortex (superior division)

## Discussion

In this study, we investigated the associations between sleep quality and visual search and attention, using a computerized task (CANTAB match-to-sample), as well as structural and functional correlates of visual attention in two attention networks (dorsal and ventral). In our sample, 43.67% of subjects reported poor sleep quality during the last month based on the PSQI. Poor sleep quality was also correlated with poor sleep hygiene and more daytime sleepiness. It was also highly associated with depression and anxiety. Despite our expectations, we found no associations between sleep quality (based on the PSQI) and visual search and attention, neither in the task nor in the imaging correlates of visual attention covarying for age, depression, and anxiety scores. However, by implementing an interaction model, we found that visual search and attention are highly associated with the DAN (both structural and functional) in the PS group and with the VAN in the GS group.

One-night lack of sleep is not uncommon among adolescents and early adulthood, and many studies have investigated its effects on cognition so far (Killgore, 2010). However, most studies instructed participants to forcefully not sleep for the purpose of their study. It was not until recently that the effects of poor sleep quality during a longer period (e.g., 1 month) are studied (Tahmasian et al., 2021). Poor sleep quality is a common finding in shift workers, such as medical and military staff (Almojali et al., 2017; Capaldi et al., 2019), but it is also prevalent in the whole population as well, which is estimated to affect one-third to half of the studied population (Hinz et al., 2017). This raises a major question: What are the neurobiological foundations of poor sleep quality in the general population? It is not unexpected to find lower sleep quality in shift workers due to lack of nighttime sleep, its disruption (e.g., a call from ward), or the stressful nature of their jobs, usually being medical or military (Almojali et al., 2017; Capaldi et al., 2019). However, what about the general population? Sleep is a complex behavior relating to psychiatric factors such as mood and anxiety levels (Ramsawh et al., 2009; Huang and Zhu, 2020). This association is as much regarding sleep-related complaints as at least one of the DSM-V criteria for both depression and anxiety disorders (American Psychiatric Association, 2013; Regier et al., 2013). Importantly, one must not confuse correlation with causality; the direction of causality between sleep disruption and psychiatric problems is not clear, but it seems that it is a bidirectional arrow: poor sleep quality can result in psychiatric problems and *vice versa* (Talbot et al., 2010; Roberts and Duong, 2014; Al-Abri, 2015; Pires et al., 2016). We also found a high correlation between poor sleep quality and both depression and anxiety. Considering this correlation and the cognitive association of these disorders (Weierich et al., 2008; de Fockert and Cooper, 2014; Gärtner et al., 2018; Mogg and Bradley, 2018; Keller et al., 2019; Berggren and Eimer, 2021), adjusting for depression and anxiety in sleep-related studies is inevitable.

Decreased sustained attention is the most reported finding of cognitive effects of short-period sleep deprivation (Gunzelmann et al., 2009; Killgore, 2010; Hudson et al., 2020). Its implications reach far from the area of cognitive sciences, and it is believed that disrupted visual attention may underlie higher rates of accidents after sleep deprivation (Wibirama et al., 2015; Wijayanto et al., 2018). However, compared to short-period wakefulness, a few studies have investigated the cognitive effects of low sleep quality over a longer period, yet visual attention itself. Siddarth et al. (2021) showed that, based on Conner’s continuous performance test (CPT), poor sleep quality was associated with decreased sustained attention in an elderly population (mean age = 60.4). Gobin et al. (2015) also reached the same results using a Go/No-Go paradigm to evaluate sustained attention. Of note, both studies evaluate sustained attention based on a task designed with either a letter or number as their stimulus and were not visual attention tasks. Being the first to assess visual attention in poor sleep quality, we did not find any associations between PSQI measures and match-to-sample reaction times, as a measure of visual attention in our sample of 34 subjects. This can be either a genuine finding or due to sample size. There are methods to further analyze negative findings; the simplest and usually used method is the two one-sided test (TOST) (Schuirmann, 1987; Lakens et al., 2018). It is a simple approach to test whether the results fall within a specified range of effect size (e.g., Cohen’s d) to conclude the absence of an effect. There is an R package called TOSTER to do it (Lakens, 2017); however, it requires the smallest effect size of interest (SESOI) to be determined and compares the results to it. Lakens et al. (2018) proposed a few methods. The first approach involves using a pre-determined fixed effect size (e.g., 0.3 or 0.5), but it is not suggested because the effect rejections may not be applicable to all studies (Hemphill, 2003; Weber and Popova, 2012). The second approach is proposed by Simonsohn (2015) based on the effect sizes of a prior study. Since our study is the first in this regard, we did not have any effect size estimation based on prior findings. Also, no differences in the visual attention task performance were replicated in the underlying imaging findings as well; there were no differences in the VAN and DAN between GSs and PSs, both in their fMRI signal and tract properties connecting their nodes. Thus, we believe that our finding is a genuine finding indicating no effects of sleep quality on visual attention, yet future studies are obviously required.

Few studies have investigated neural correlates of poor sleep quality in a healthy population. In a study by Amorim et al. (2018), they implemented a whole-brain functional and diffusion MRI approach toward neural association with poor sleep quality in an elderly population. As in our study on the younger population, they had no significant findings in the white matter properties (i.e., FA, MD, AD, and RD); however, they found lower functional connectivity in a network comprising lower parietal, frontal, temporal, supramarginal, insular, and Rolandic operculum regions associated with higher PSQI scores. Interestingly, they also found no regional volumetric correlates of sleep quality. The presence of no significant imaging correlates of sleep quality does not involve attention networks. Another study using an emotion task fMRI also found no association between sleep quality and neural circuits involved in emotion regulation (Minkel et al., 2012). Other studies on imaging correlates of sleep quality based on the PSQI were carried out in a population with other major underlying conditions that can affect their sleep, such as Parkinson’s disease (Gama et al., 2010), HIV+ (Venkataraman et al., 2021), or primary insomnia (Li et al., 2016).

The results of our interaction analysis are interesting findings. Our findings using both fMRI and tractography showed that task performance is highly reliant on resting activity and properties of fibers connecting the DAN (SLF1 and lateral occipital cortex) in the poor sleeper and the VAN (SLF2 and SLF3 and TPJ) in the good sleeper group. It is a multi-modal finding, found using both fMRI and DWI analysis. Despite not explicitly and overtly implementing a serial visual search task, we believe that this finding probably reflects a higher load on the DAN in PSs and not using the VAN by this group for visual search. Also, since it seems that the serial and parallel search strategies are more reliant on the DAN and VAN, respectively (Wang et al., 2020; Ischebeck et al., 2021; Slama et al., 2021), this finding indicates employing the serial visual search strategy in PSs. Importantly, the exact localization of the TPJ as an important node of the VAN is controversial; however, the supramarginal gyrus is considered a major node of TPJ (Uddin, 2015; Bernard et al., 2020).

Although our study investigates the association between sleep quality and visual search and attention in a task, as well as functional and structural connections of the attention network, it is faced with limitations. The major limitation is the sample size of 39 subjects. Nonetheless, we limited our sample to only include right-handed male subjects to decrease the between-subject variability in the brain and behavior. However, future studies must use a larger sample size including both major genders. For example, we are aware that the ENIGMA-Sleep study is a multi-center study currently conducted to overcome the limitations of small-sample studies (Tahmasian et al., 2021). Moreover, based on our interaction analysis findings, using tasks designed to separately investigate serial and parallel search strategies may better elucidate our findings. However, the MTS task does not clearly separate whether subjects have used a serial or parallel search strategy. It has a great potential in separating movement time from reaction time, being computerized and easy to do, and also estimating the increase in cognitive load by increasing the presented stimuli from 2 to 8 objects (Robbins et al., 1994), but adding an eye-tracking device or even developing it to be observed under an fMRI scanner can clearly improve its power. The readers must also bear in mind the limitations of diffusion-weighted imaging. There are high numbers of cross-fiber in the white matter, estimated to be nearly 90% of the total fibers (Jones et al., 2013; Jeurissen et al., 2014). Thus, applying multi-shell DWI sequences to higher order models is usually suggested, which can better solve the cross-fiber issue. However, using a b-value of 1,000 s/mm^2^ and a total diffusion direction of 64, as well as the recent TRACULA method, for our analysis can somehow address the cross-fiber issue.

In conclusion, to our knowledge, this study is the first to investigate visual attention and search in chronic poor sleep. Significant associations of sleep quality were found neither in a visual search and attention task nor in dorsal and ventral attention networks and fibers connecting them. However, we found that task performance is highly reliant on the DAN in the PS group and on the VAN in the GS group, probably reflecting higher cognitive demand in the DAN or implementing different search strategies (serial rather than parallel) in subjects with poor sleep. Future studies with larger sample sizes using separate serial and parallel visual search task designs are suggested.

## Data Availability Statement

The raw data supporting the conclusions of this article will be made available by the authors, without undue reservation.

## Ethics Statement

The studies involving human participants were reviewed and approved by the Ethics Review Board of the Iran University of Medical Sciences, Tehran, Iran. The patients/participants provided their written informed consent to participate in this study.

## Author Contributions

AA contributed to the design of the study, data collection, imaging analysis, statistical analysis, and wrote the manuscript. SN contributed to idea formation, designing the study, data collection, and wrote the manuscript. Both authors contributed to the article and approved the submitted version.

## Conflict of Interest

The authors declare that the research was conducted in the absence of any commercial or financial relationships that could be construed as a potential conflict of interest.

## Publisher’s Note

All claims expressed in this article are solely those of the authors and do not necessarily represent those of their affiliated organizations, or those of the publisher, the editors and the reviewers. Any product that may be evaluated in this article, or claim that may be made by its manufacturer, is not guaranteed or endorsed by the publisher.

## Footnotes

1
www.nitrc.org/projects/conn

2
https://trendscenter.org/software/gift/
