# Supplementary material for: Visual Attention and Poor Sleep Quality
Source: Front Neurosci. 2022 Jun 2;16:850372. doi: 10.3389/fnins.2022.850372 (PMC9202476; doi:10.3389/fnins.2022.850372)
Supplement: Supplementary file 1 [file Table_1.DOCX]

| Supplementary Table 1. Estimate (Std. Error) of general linear models with match-to-sample (MTS) task parameters as outcome, Pittsburgh Sleep Quality Index (PSQI) total score and components as predictors, and age, depression, and anxiety scores as covariates. RT: Reaction time. | | | |
| --- | --- | --- | --- |
| MTS Parameter | PSQI Variable | Estimate (Std. Error) | *p*-value |
| Total Correct | Total Score | -0.031 (0.146) | 0.832 |
| Total Correct | Component 1 | 0.341 (0.553) | 0.542 |
| Total Correct | Component 2 | -0.499 (0.367) | 0.185 |
| Total Correct | Component 3 | 0.201 (0.523) | 0.704 |
| Total Correct | Component 4 | 0.152 (0.643) | 0.815 |
| Total Correct | Component 5 | -0.16 (0.64) | 0.804 |
| Total Correct | Component 6 | -0.482 (1.307) | 0.715 |
| Total Correct | Component 7 | 0.092 (0.44) | 0.836 |
| Mean Correct RT | Total Score | -43.816 (51.39) | 0.401 |
| Mean Correct RT | Component 1 | 64.869 (198.296) | 0.746 |
| Mean Correct RT | Component 2 | -32.9 (135.208) | 0.81 |
| Mean Correct RT | Component 3 | 4.043 (187.355) | 0.983 |
| Mean Correct RT | Component 4 | -269.727 (223.941) | 0.238 |
| Mean Correct RT | Component 5 | -119.313 (227.541) | 0.604 |
| Mean Correct RT | Component 6 | -724.307 (447.066) | 0.116 |
| Mean Correct RT | Component 7 | -134.075 (155.154) | 0.395 |
| Mean RT Change (2 to 8) | Total Score | -117.843 (82.454) | 0.164 |
| Mean RT Change (2 to 8) | Component 1 | 84.438 (325.594) | 0.797 |
| Mean RT Change (2 to 8) | Component 2 | -216.698 (218.274) | 0.329 |
| Mean RT Change (2 to 8) | Component 3 | 80.653 (307.036) | 0.795 |
| Mean RT Change (2 to 8) | Component 4 | -383.345 (369.81) | 0.309 |
| Mean RT Change (2 to 8) | Component 5 | -543.904 (360.821) | 0.143 |
| Mean RT Change (2 to 8) | Component 6 | -1478.599 (714.457) | 0.048 |
| Mean RT Change (2 to 8) | Component 7 | -288.328 (252.127) | 0.262 |

| Supplementary Table 2. Interaction result parameters (Estimate (Std. Error)) and *p*-values of general linear models with match-to-sample (MTS) scores as outcome, and group × fiber properties as predictor variables. Age, depression, and anxiety scores were covaried. For statistical inference of the table below, we have to indicate that we defined group in R as a factor variable with poor sleep = 1 and good sleep = 0 (PSQI cut-off > 5). RT: Reaction Time, SLF: Superior Longitudinal Fasciculus, AF: Arcuate Fasciculus, FA: Fractional Anisotropy, AD: Axial Diffusivity, MD: Mean Diffusivity, RD: Radial Diffusivity. | | | | |
| --- | --- | --- | --- | --- |
| MTS Score | Tract Properties | Estimate | Std. Error | *p*-value |
| Total Correct | Left SLF1 AD | -18189.52 | 12793.74 | 0.169 |
| Total Correct | Left SLF1 FA | 6.33 | 29.30 | 0.831 |
| Total Correct | Left SLF1 MD | -27183.81 | 28972.65 | 0.358 |
| Total Correct | Left SLF1 RD | -26679.95 | 28175.53 | 0.354 |
| Total Correct | Left SLF2 AD | -32342.09 | 29801.55 | 0.290 |
| Total Correct | Left SLF2 FA | -10.42 | 36.44 | 0.777 |
| Total Correct | Left SLF2 MD | -12782.29 | 42026.24 | 0.764 |
| Total Correct | Left SLF2 RD | -3309.18 | 33674.24 | 0.923 |
| Total Correct | Left SLF3 AD | -32225.19 | 21144.45 | 0.142 |
| Total Correct | Left SLF3 FA | -20.50 | 31.56 | 0.523 |
| Total Correct | Left SLF3 MD | -9531.87 | 32963.30 | 0.775 |
| Total Correct | Left SLF3 RD | 5798.75 | 30535.05 | 0.851 |
| Total Correct | Right AF AD | -45447.62 | 28512.23 | 0.125 |
| Total Correct | Right AF FA | -5.33 | 36.20 | 0.884 |
| Total Correct | Right AF MD | -41178.12 | 41687.89 | 0.334 |
| Total Correct | Right AF RD | -13011.86 | 34078.56 | 0.706 |
| Total Correct | Right SLF1 AD | -18734.38 | 19230.88 | 0.341 |
| Total Correct | Right SLF1 FA | 16.79 | 31.27 | 0.597 |
| Total Correct | Right SLF1 MD | -46957.30 | 31599.09 | 0.151 |
| Total Correct | Right SLF1 RD | -31241.39 | 27585.07 | 0.270 |
| Total Correct | Right SLF2 AD | -37741.04 | 32139.38 | 0.253 |
| Total Correct | Right SLF2 FA | 14.08 | 36.80 | 0.706 |
| Total Correct | Right SLF2 MD | -38830.46 | 39893.75 | 0.341 |
| Total Correct | Right SLF2 RD | -20467.61 | 32357.55 | 0.534 |
| Total Correct | Right SLF3 AD | -60588.25 | 26261.32 | 0.031* |
| Total Correct | Right SLF3 FA | -39.25 | 39.83 | 0.335 |
| Total Correct | Right SLF3 MD | -55076.21 | 42005.13 | 0.203 |
| Total Correct | Right SLF3 RD | -5391.84 | 40360.60 | 0.895 |
| Mean RT Change (2 to 8) | Left SLF1 AD | -17321725.49 | 9454483.88 | 0.081 |
| Mean RT Change (2 to 8) | Left SLF1 FA | 74.41 | 19385.05 | 0.997 |
| Mean RT Change (2 to 8) | Left SLF1 MD | -30197792.05 | 19891511.95 | 0.143 |
| Mean RT Change (2 to 8) | Left SLF1 RD | -8025298.60 | 18753205.55 | 0.673 |
| Mean RT Change (2 to 8) | Left SLF2 AD | 1390159.93 | 21812858.33 | 0.950 |
| Mean RT Change (2 to 8) | Left SLF2 FA | 30704.66 | 23235.18 | 0.200 |
| Mean RT Change (2 to 8) | Left SLF2 MD | -24595855.56 | 28817501.52 | 0.403 |
| Mean RT Change (2 to 8) | Left SLF2 RD | -25068254.09 | 22048882.06 | 0.268 |
| Mean RT Change (2 to 8) | Left SLF3 AD | -5551573.80 | 15810607.25 | 0.729 |
| Mean RT Change (2 to 8) | Left SLF3 FA | 22228.19 | 20141.27 | 0.282 |
| Mean RT Change (2 to 8) | Left SLF3 MD | -23862035.13 | 22643033.21 | 0.303 |
| Mean RT Change (2 to 8) | Left SLF3 RD | -23216686.04 | 19746192.28 | 0.252 |
| Mean RT Change (2 to 8) | Right AF AD | 17984340.86 | 19529998.63 | 0.367 |
| Mean RT Change (2 to 8) | Right AF FA | 45239.70 | 21349.08 | 0.046* |
| Mean RT Change (2 to 8) | Right AF MD | -12179894.51 | 28178504.60 | 0.670 |
| Mean RT Change (2 to 8) | Right AF RD | -25014317.97 | 21835872.02 | 0.264 |
| Mean RT Change (2 to 8) | Right SLF1 AD | -16137266.05 | 12976478.74 | 0.227 |
| Mean RT Change (2 to 8) | Right SLF1 FA | 564.43 | 19989.55 | 0.978 |
| Mean RT Change (2 to 8) | Right SLF1 MD | -23760494.73 | 21923272.26 | 0.290 |
| Mean RT Change (2 to 8) | Right SLF1 RD | -9906938.47 | 18445916.33 | 0.597 |
| Mean RT Change (2 to 8) | Right SLF2 AD | 17959108.09 | 21638141.09 | 0.415 |
| Mean RT Change (2 to 8) | Right SLF2 FA | 49406.27 | 22176.81 | 0.036* |
| Mean RT Change (2 to 8) | Right SLF2 MD | -19856443.38 | 26673324.38 | 0.464 |
| Mean RT Change (2 to 8) | Right SLF2 RD | -29050178.87 | 20789405.36 | 0.176 |
| Mean RT Change (2 to 8) | Right SLF3 AD | 4686219.08 | 19292987.31 | 0.810 |
| Mean RT Change (2 to 8) | Right SLF3 FA | 23779.77 | 25363.30 | 0.359 |
| Mean RT Change (2 to 8) | Right SLF3 MD | -19021679.41 | 28153033.34 | 0.506 |
| Mean RT Change (2 to 8) | Right SLF3 RD | -16735142.59 | 25480041.15 | 0.518 |
| Mean Correct RT | Left SLF1 AD | -11864398.88 | 5445317.86 | 0.040* |
| Mean Correct RT | Left SLF1 FA | -4096.87 | 11556.84 | 0.726 |
| Mean Correct RT | Left SLF1 MD | -15285392.86 | 11813929.50 | 0.209 |
| Mean Correct RT | Left SLF1 RD | -416057.75 | 11053061.08 | 0.970 |
| Mean Correct RT | Left SLF2 AD | -1744866.75 | 12987278.22 | 0.894 |
| Mean Correct RT | Left SLF2 FA | 19075.54 | 13705.35 | 0.178 |
| Mean Correct RT | Left SLF2 MD | -17306095.49 | 16979788.48 | 0.319 |
| Mean Correct RT | Left SLF2 RD | -15961924.75 | 12981546.35 | 0.232 |
| Mean Correct RT | Left SLF3 AD | -2103021.76 | 9421760.08 | 0.825 |
| Mean Correct RT | Left SLF3 FA | 14602.22 | 11673.09 | 0.224 |
| Mean Correct RT | Left SLF3 MD | -12344404.04 | 13307973.97 | 0.364 |
| Mean Correct RT | Left SLF3 RD | -13078499.19 | 11465954.72 | 0.266 |
| Mean Correct RT | Right AF AD | 10425426.38 | 11646111.25 | 0.380 |
| Mean Correct RT | Right AF FA | 27439.77 | 12884.03 | 0.045* |
| Mean Correct RT | Right AF MD | -7051199.46 | 16555394.89 | 0.674 |
| Mean Correct RT | Right AF RD | -16022113.84 | 12867143.06 | 0.226 |
| Mean Correct RT | Right SLF1 AD | -6773847.52 | 7902403.60 | 0.401 |
| Mean Correct RT | Right SLF1 FA | 1145.58 | 11747.51 | 0.923 |
| Mean Correct RT | Right SLF1 MD | -9569804.03 | 13123474.14 | 0.474 |
| Mean Correct RT | Right SLF1 RD | -3909546.58 | 10836590.14 | 0.722 |
| Mean Correct RT | Right SLF2 AD | 10845047.86 | 12870352.25 | 0.408 |
| Mean Correct RT | Right SLF2 FA | 28411.06 | 13324.30 | 0.044* |
| Mean Correct RT | Right SLF2 MD | -10855430.31 | 15800851.53 | 0.499 |
| Mean Correct RT | Right SLF2 RD | -17156908.38 | 12321199.20 | 0.178 |
| Mean Correct RT | Right SLF3 AD | 441080.29 | 11538223.33 | 0.970 |
| Mean Correct RT | Right SLF3 FA | 14090.25 | 15376.99 | 0.369 |
| Mean Correct RT | Right SLF3 MD | -11956228.58 | 16556449.63 | 0.478 |
| Mean Correct RT | Right SLF3 RD | -10087887.63 | 15124671.05 | 0.512 |
| ^*.^ *^p­^*^-value < 0.05.^ | | | | |
